# Interaction of ferroptosis and cuproptosis in the perspective of pulmonary hypertension

**DOI:** 10.3389/fcvm.2025.1611449

**Published:** 2025-06-26

**Authors:** Yan Yang, Lu Liang, Wanjuan Pei, Yinhui Sun

**Affiliations:** School of Medicine, Hunan University of Chinese Medicine, Changsha, Hunan, China

**Keywords:** ferroptosis, cuproptosis, pulmonary hypertension, oxidative stress, metabolic reprogramming, mitochondrial dysfunction

## Abstract

Copper (Cu) and iron (Fe) are essential trace elements that are involved in normal human metabolic processes. Disruption of their homeostasis contributes to disease pathogenesis through mechanisms such as cuproptosis and ferroptosis. Cuproptosis targets lipoylated proteins to disrupt mitochondrial respiration, whereas ferroptosis is driven by lipid peroxidation. These processes may independently or interactively exacerbate pulmonary hypertension (PH), a condition characterized by progressive pulmonary vascular remodeling, clinical manifestations of dyspnea, right-sided heart failure, and high mortality, via oxidative stress, metabolic reprogramming, and other mechanisms. This review systematically elucidates: (1) the updated molecular mechanisms of cuproptosis/ferroptosis, (2) research evidence for their roles in PH, and (3) synergistic crosstalk in different subtypes of PH progression. We propose that coordination and regulation of the crosstalk network between cuproptosis and ferroptosis may represent a novel therapeutic strategy for pulmonary vascular remodeling.

## 1 Introduction

Pulmonary hypertension (PH) is a cardiopulmonary disorder characterized by an elevated mean pulmonary arterial pressure (mPAP) of ≥20 mmHg at rest, accompanied by abnormal pulmonary vascular pressure. Hemodynamically, precapillary PH is defined by mPAP ≥20 mmHg, pulmonary vascular resistance (PVR) ≥3 Wood units, and pulmonary artery wedge pressure (PAWP) ≤15 mmHg (1), whereas postcapillary PH requires mPAP ≥20 mmHg with PAWP >15 mmHg (2). The World Health Organization classifies PH into five groups: pulmonary arterial hypertension (PAH), PH because of left heart disease (PH-LHD), PH associated with lung diseases/hypoxia (PH-LD), Chronic Thromboembolic Pulmonary Hypertension (CTEPH), and PH of unclear multifactorial mechanisms (2, 3). Recent epidemiological studies revealed an aging-related rise in PH incidence among industrialized nations, affecting approximately 1% of the global population—including ≥50% of heart failure patients, 10% of chronic obstructive pulmonary disease (COPD) cases, and 10% of adults aged >65 years (4, 5).

Although vasodilatory therapies (6) and initial combination strategies [e.g., dual targeting of the endothelin and nitric oxide pathways with the endothelin receptor antagonist macitentan and phosphodiesterase type 5 inhibitor (PDE5i) tadalafil] have demonstrated efficacy in arterial and thromboembolic PH, challenges persist. Notably, such combination therapy significantly reduces PVR compared to monotherapy but concurrently increases severe adverse events (7), underscoring the urgent need for novel pathway exploration to curb disease progression.

Recent studies have identified nonapoptotic cell death pathways as promising therapeutic targets (8). Ferroptosis, first proposed by Dixon in 2012 (9), correlates with PH severity (10) through iron (Fe) accumulation-driven Fenton reactions that generate oxygen radicals, induce lipid peroxidation, and disrupt membrane integrity. While physiologically involved in embryogenesis, ferroptosis also exhibits therapeutic potential in oncology, cardiovascular, and gastrointestinal disorders (11, 12). Recently, Tsvetkov et al. (13) identified cuproptosis, a ferredoxin 1(FDX1)-dependent process that disrupts mitochondrial respiration via the modulation of lipoylated proteins and iron-sulfur (Fe-S) proteins. This discovery has expanded research in the fields of cancer, neurology, and cardiovascular diseases (14, 15). Notably, cuproptosis and ferroptosis may synergistically exacerbate PH progression through mutual reinforcement, and their co-targeting strategies have shown preclinical promise in oncology and immunotherapy (16) (Table 1).

**Table 1:** Ferroptosis-related factors.

Abbreviation	Full name	Function	References
ACSL4	Acyl-CoA synthetase long-chain family member 4	Catalyzes the generation of CoA derivatives from polyunsaturated fatty acids, promotes lipid peroxidation, and drives ferroptosis	(17–19)
GPX4	Glutathione peroxidase 4	Core antioxidant enzymes. Inhibits ferroptosis by reducing lipid hydroperoxides and maintains redox homeostasis	(20, 21)
SLC7A11	Solute carrier family 7 member 11	Forms the Xc^−^ system, mediating cystine uptake for glutathione synthesis to inhibit ferroptosis	(22, 23)
SLC7A5	Solute carrier family 7 member 5	Transports cystine, thereby promoting glutathione synthesis and reducing lipid peroxidation levels	(22–24)
FSP1	Ferroptosis suppressor protein 1	Reduces coenzyme Q10 and vitamin K to synergistically inhibit ferroptosis with GPX4	(20)
TFR1	Transferrin receptor 1	Mediates iron uptake into cells via transferrin endocytosis, promoting iron accumulation	(25, 26)
4-HNE	4-Hydroxynonenal	Biomarkers of ferroptosis. Covalently binds to GPX4, inactivating it and thereby exacerbating lipid peroxidation	(27, 28)
CoQ10	Coenzyme Q10	Inhibits lipid peroxidation by reducing coenzyme Q10 through FSP1, thereby protecting cells from ferroptosis	(20, 29)
PRDX3	Peroxiredoxin 6	Inhibits cystine uptake to trigger ferroptosis; translocates to the plasma membrane after peroxidation	(30)
HMGB1	High Mobility Group Box 1	In ferroptosis, it is released to activate the TLR4/NLRP3 inflammasome pathway, thereby exacerbating inflammation	(31)

## 2 Ferroptosis

### 2.1 The concept of ferroptosis

Ferroptosis is a newly identified form of cell death that depends on iron ions and lipid peroxidation (20). Current research on its mechanisms relies on the detection of key biomarkers, such as the lipid peroxidation-related molecules Acyl-CoA Synthetase Long-Chain Family Member 4 (ACSL4) and Arachidonate 15-Lipoxygenase, as well as the dynamic changes in the lipid peroxidation products 4-Hydroxy-2-Nonenal (4-HNE) and malondialdehyde (17, 18). Lipid peroxidation involves two mechanisms: (1) the active site of lipoxygenase contains Fe^3+^, which catalyzes the oxidation of polyunsaturated fatty acids (PUFAs), and (2) polyunsaturated fatty acids undergo oxidation reactions with reactive oxygen species (ROS) and Fe^2+^. Both these processes can generate lipid hydroperoxides (LOOH), which ultimately participate in ferroptosis, The hallmark of ferroptosis is the inhibition of glutathione peroxidase 4 (GPX4), which can reduce LOOH to maintain cellular homeostasis (21). Additionally, ferroptosis suppressor protein 1 (FSP1) selectively inhibits ferroptosis by targeting the plasma membrane, thereby exerting a protective effect (20). These core regulatory mechanisms focus on the role of the lipid metabolism network in the maintenance of plasma membrane integrity. As the molecular mechanisms of ferroptosis are further elucidated, therapeutic strategies targeting this process may open new avenues for the treatment of related diseases.

### 2.2 Mechanisms of ferroptosis

#### 2.2.1 The Xc^−^ system

Ferroptosis is closely related to the Xc ^−^ system activity. This system is composed of serum solute carrier family 7 member 11 (SLC7A11) and solute carrier family 3 member 2 (SLC3A2) subunits, which mediate the exchange of cysteine and glutamate and provide cysteine for glutathione synthesis. Notably, impairment of the Xc^−^ System leads to glutathione deficiency, which in turn results in decreased GPX4 activity, contributing to ferroptosis (22).

Solute carrier family 7 member 5 (SLC7A5), a component subunit of SLC3A2, can be induced by interleukin-3 (24) and plays a crucial role in the function of the Xc^−^ system (32). Sulfasalazine and erastin (Er) promote ferroptosis by inhibiting the Xc^−^ system: Er, as a small-molecule activator of ferroptosis, binds to SLC7A5, indirectly preventing cystine uptake, while Ras-selective lethal small molecule 3 (RSL3) covalently binds to GPX4. Both of these actions inhibit the function of the Xc^−^ system, thereby regulating ferroptosis (23). Compared to GPX4, RSL3, and ML162, two ferroptosis inducers more directly inhibit selenoprotein thioredoxin reductase 1 (TXNRD1), which clears peroxides (33).

#### 2.2.2 Lipid oxidation

Fatty acids are critical components of cell membranes and signal transduction pathways. Peroxidation of PUFAs is central to ferroptosis. Lipid peroxidation typically requires a combination of peroxyl radicals (RO•) and hydroxyl radicals (HO•) with fatty acids (34). The redox cycling of Fe³^+^/Fe²^+^ in the Haber–Weiss reaction and the Fe²^+^-mediated Fenton reaction both produce large amounts of ROS. PUFAs, such as arachidonic acid (AA) and adrenic acid (AdA), are prone to lipid peroxidation by ROS because of their bis-allylic hydrogen atoms. This process is catalyzed by ACSL4, which converts PUFAs into Coenzyme A derivatives, which are esterified into phosphatidylethanolamines (PEs) by lysophosphatidylcholine acyltransferase 3. These PEs, rich in easily oxidizable AA/AdA, disrupt the lipid bilayer, leading to cell death and participating in pathological processes such as aortic endothelial cell atherosclerosis (19, 35). Ma et al. (9) showed that the binding of the 3′ untranslated region of ACSL4 with miR-424-5p can suppress ACSL4 expression, thereby alleviating ferroptosis induced by Erastin and RSL3.

#### 2.2.3 Fe metabolism

Disorders in Fe metabolism are closely linked to ferroptosis. Dietary Fe exists in two forms: Fe²^+^ and Fe³^+^. Fe³^+^, which is poorly soluble in water, must bind to proteins for transport to participate in the synthesis of erythrocytes and other Fe-dependent cells. Once bound to transferrin (TF), Fe³^+^ is internalized via receptor-mediated endocytosis through transferrin receptor 1 and subsequently reduced to Fe²^+^ by metalloreductase STEAP3, Fe²^+^, which is highly water-soluble and reactive, exerts cytotoxic effects. It is then transported into the cytosolic labile Fe pool via divalent metal transporter 1 (DMT1) (25, 36). Ferritin, composed of ferritin light chain 1 (FTL1) and ferritin heavy chain 1 (FTH1), stores Fe³^+^ after FTH1 converts Fe²^+^. Ferritin can be released via exosomes to enable Fe efflux. When Fe²^+^ accumulates excessively, ferritin interacts with NCOA4, undergoes autophagy, and releases Fe²^+^ within the cell, promoting lipid peroxidation and triggering ferroptosis (26). Cytosolic Fe²^+^ is extruded through ferroportin 1 (FPN1) on the basolateral membrane, oxidized to Fe³^+^ by the membrane Fe oxidase hephaestin (HEPH), and then binds to TF to enter the circulation. Hepcidin binds to ferroportin to inhibit Fe²^+^ efflux, whereas DMT1 promotes Fe²^+^ uptake. These processes collectively maintain Fe homeostasis (37, 38). Experimental evidence (39, 40) shows that Fe-responsive element-binding protein 2 (IREB2) enhances its stability by binding to the deubiquitinase OTU Deubiquitinase 1, which promotes IREB2 deubiquitination. This increases the expression of downstream genes, Transferrin Receptor and DMT1, leading to Fe²^+^ accumulation and facilitating ferroptosis. In summary, Fe metabolism imbalance drives ferroptosis via multiple pathways.

## 3 Cuproptosis

### 3.1 Definition and discovery of cuproptosis

Maintaining Copper (Cu) ion homeostasis is crucial for human health. Cu can primarily exist in two oxidative states: Cu^+^, which accounts for 95% of the total intracellular Cu content, and Cu^2+^, which constitutes 5% of extracellular Cu. The reduction of Cu²^+^ in the gut to Cu^+^ by metal reductases is followed by Cu uptake in a Ctr1-dependent manner. Notably, the form of Cu(copper complex or copper ions) also affects the accumulation efficiency (41, 42). Following absorption, Cu is exported into the bloodstream via the P-type ATPases ATP7A and ATP7B (43). Cu^+^ in the blood is easily oxidized to Cu²^+^, which can bind with plasma proteins, among which Ceruloplasmin (CP) transports Cu²^+^ to various organs. However, Intracellular Cu^+^ is delivered to specific targets by chaperones such as antioxidant 1 copper chaperone (Atox1), cytochrome c oxidase copper chaperone (Cox17), and copper chaperone for superoxide dismutase (CCS), or it can be stored in metallothionein (44). Dysregulated Cu metabolism has been implicated in metabolic disorders, providing a rationale for targeting Cu homeostasis in therapies (14, 45). Even before the formal concept of cuproptosis was proposed, studies showed that Cu imbalance could suppress cytochrome c oxidase (COX) activity, impair mitochondrial membrane potential, reduce energy production, activate the AMP-activated protein kinase (AMPK) pathway, shift cellular metabolism toward glycolysis, and inhibit cell growth (46, 47). In 2022, researchers discovered that Cu ions, when delivered by carriers such as elesclomol, bind to lipoylated proteins in the tricarboxylic acid (TCA) cycle. The intracellular reductase FDX1 promotes the synthesis of lipoic acid synthase (LIAS), which regulates lipoylation. Under Cu overload conditions, reduced FDX1 specifically interacts with the Cu ionophore elesclomol or elesclomol-Cu(II), facilitating the conversion of excess Cu²^+^ into more toxic Cu^+^ (48, 49). The released Cu^+^ subsequently binds to lipoylated proteins, inducing proteotoxic stress and ultimately leading to cell death (50). This process, termed cuproptosis, is accompanied by the loss of Fe-S cluster proteins. This form of cell death is uniquely reversible by Cu chelators, distinguishing it from other regulated cell death pathways (Table 2).

**Table 2:** Cuproptosis-related factors.

Abbreviation	Full name	Function	References
FDX1	Ferredoxin 1	Cuproptosis core regulatory factor. mediating copper ion-dependent cell death by modulating Lipoylated proteins and iron-sulfur cluster proteins	(13, 45)
LIAS	Lipoic acid synthetase	The necessary condition for cuproptosis. Catalyzing the conjugation of lipoic acid with target proteins to generate Lipoylation	(51, 52)
DLAT	Dihydrolipoamide Acetyltransferase	Lipoylated target proteins. Inducing mitochondrial proteotoxic stress through oligomerization during copper overload	(13, 53)
DLST	Dihydrolipoamide S-succinyltransferase	The components of the key enzyme α-ketoglutarate dehydrogenase complex in the tricarboxylic acid cycle. Binding to copper leads to loss of its own function, thereby inhibiting energy metabolism	(13, 54)
GLS	Glutaminase	Anti-cuproptosis genes. Maintaining glutamine metabolism, alleviate copper-induced mitochondrial dysfunction.	(55)
MTF1	Metal-regulatory transcription factor 1	Anti-cuproptosis genes. Activating metallothioneins to chelate excess copper when cells encounter excess copper	(53)
SLC31A1	Solute carrier family 31 member 1	Mediating copper ion uptake. Promoting cuproptosis under copper overload conditions	(39, 56)
PDH	Pyruvate Dehydrogenase Complex	The key enzyme linking glycolysis and the tricarboxylic acid cycle (TCA cycle). Inhibition of activity during cuproptosis affects mitochondrial energy metabolism	(53, 57)
ATOX1	Antioxidant 1 Copper Chaperone	Copper chaperone proteins. Regulates copper transport and SOD expression, It can alleviate oxidative stress	(59, 60)

### 3.2 Mechanisms of cuproptosis

#### 3.2.1 Mitochondrial mechanism: targeting the TCA cycle

Cu-induced cell death has been extensively studied over the past decade (61). Tsvetkov et al. first demonstrated its effect on mitochondrial respiration (13). Mitochondria serve as key hubs for energy metabolism and apoptotic signaling and harbor a Cu reservoir. Excessive Cu accumulation disrupts mitochondrial enzyme activity and impairs respiration (62). Intracellular Cu^+^, a cofactor for COX and SOD1, can have its deficiency restored by Cu ionophores to regain enzyme activity. This genetic disorder is caused by pathogenic mutations in the Cu transporter ATP7A, leading to systemic Cu deficiency. Cu ionophores have also shown potential for therapeutic applications (63), among which SOD1 has antioxidant functions. Cu^+^ binds to SOD1 and promotes the formation of its disulfide (-S-S-) bonds through the mediation of the CCS (64). In addition, the synthesis of cytochrome c oxidase (SCO) proteins significantly influences the maintenance of Cu ion homeostasis within the mitochondria. For instance, the mitochondrial cytochrome C oxidase Cu chaperone and SCO2 mediate Cu transport into the mitochondria and its allocation to COX (65, 66). Oxidized cysteine residues in mitochondrial SCO1 bind Cu^+^, generating redox signals transmitted via cytochrome c oxidase assembly factor 19 to cytosolic ATP7A, promoting Cu efflux (67). Furthermore, Cu forms complexes with ligands in the mitochondrial matrix, interfering with FDX2-dependent Fe-S cluster protein maturation and exacerbating mitochondrial dysfunction (54–52). Thus, Cu dyshomeostasis poses a severe threat to mitochondrial integrity.

Several enzymes involved in mitochondrial metabolism are associated with apoptosis. Cu-induced cell death depends on protein lipoylation, which is a post-translational modification of lysine residues. First described in the 1960s, lipoylation occurs in only five proteins: dihydrolipoamide acetyltransferase (DLAT), pyruvate dehydrogenase complex componentX(PDHX), dihydrolipoamide succinyltransferase (DLST), dihydrolipoamide branched-chain transacylase, and glycine cleavage system protein H (58). FDX1 directly binds to LIAS and provides electrons to enable LIAS-catalyzed reactions. Therefore, upon LIAS knockdown, the level of lipoylation decreases (53–69). Consequently, in Cu overload conditions, Cu^+^ binds to acylated DLAT and DLST, inducing their oligomerization and depleting Fe-S cluster proteins, leading to cell death in an FDX1-dependent manner (13). Recent studies have identified seven pro-cuproptosis genes [*FDX1*, *LIAS*, *lipoyltransferase 1 (LIPT1), DLD*, *DLAT*, *PDHA1*, and *PDHB*] and three anti-cuproptosis genes [*MTF1*, *Glutaminase(GLS*), and *CDKN2A*], Notably, the pyruvate dehydrogenase (PDH) complex plays a central role in these pathways (70), highlighting its critical role in the TCA cycle during cuproptosis.

#### 3.2.2 Cu-induced oxidative stress

Cu-induced oxidative stress plays a pivotal role in disease progression. The TCA cycle, a primary site for ROS generation, is targeted by Cu ions, leading to cuproptosis, suggesting that oxidative stress is an inevitable consequence of cuproptosis. Moreover, erythroid 2-related factor 2 (Nrf2), a central regulator of oxidative stress, is activated by Cu overload to initiate autophagy and antioxidant responses (71). This process can be rescued by Cu chelators, such as tetrathiomolybdate (TTM), potentially through the AMPK/mTOR/ULK1 pathway-mediated degradation of Kelch-like ECH-associated protein 1(KEAP1), a negative regulator of Nrf2 (72). This indicates that Cu overload can exert oxidative stress via Nrf2, and that Cu-induced oxidative stress damage may involve both apoptosis and autophagy (59). Autophagy, a process of cellular self-digestion and degradation (60), has been shown to ameliorate the progression of pulmonary arterial hypertension (73), suggesting that an imbalance in Cu homeostasis may exacerbate disease progression through the Nrf2-autophagy-oxidative stress axis. In addition, within the vasculature, the generation of ROS can occur not only through the reduction of Cu^2+^ to Cu^+^, but also via the ATOX1-TRAF axis. This process can disrupt Fe-S clusters and induce damage to vascular endothelial and smooth muscle cells, which has been demonstrated in cardiovascular pathologies (74). Antioxidant 1, a chaperone protein for Cu^+^, maintains the redox balance by regulating Cu transport and superoxide dismutase [SOD])expression. A reduction in ATOX1 can trigger oxidative stress (75) and chelating Cu ions can mitigate this oxidative stress (30, 31). These intricate interactions not only highlight that dysregulation of Cu metabolism induces oxidative stress through multiple pathways, but also underscore the potential of targeting these pathways as novel therapeutic strategies for cardiovascular diseases. Therefore, the molecular mechanisms underlying these processes require further investigation.

## 4 Therapeutic potential of cuproptosis and ferroptosis research

Pulmonary arterial remodeling, which leads to increased vascular pressure, often results in secondary myocardial dysfunction, which progressively develops into heart failure, ultimately resulting in mortality. Both cuproptosis and ferroptosis have significant potential in this context.

### 4.1 Ferroptosis as a potential therapeutic target in PH

In PH, ferroptosis often exacerbates disease progression, primarily through a lipid peroxidation imbalance, which disrupts the redox equilibrium in pulmonary vascular endothelial and smooth muscle cells. In the vascular endothelium, promotion of the binding of SLC3A2 to ubiquitin induces ferroptosis, thereby causing endothelial injury. Similarly, PAH endothelial cells often exhibit pro-ferroptotic phenotypes. For instance, the overexpression of ACSL4, which is involved in lipid peroxidation, in PAECs induces ferroptosis, leading to an inflammatory pulmonary vascular burden, triggering PAH and subsequently inducing right ventricular dysfunction (10, 76). Additionally, peroxiredoxin 3 (PRDX3) serves as a crucial antioxidant protein. After peroxidation, it translocates from the mitochondria to the plasma membrane, where it triggers ferroptosis by inhibiting cystine uptake (77). Based on this, Liao et al. (78) found that peroxiredoxin 6, which belongs to the same family as that of PRDX3, can prevent the release of high-mobility group box 1 (HMGB1) induced by ferroptosis in PAECs and inhibit the TLR4/NLRP3 inflammasome pathway, thereby alleviating inflammatory responses and reversing PH. Similarly, Xie et al. (79) demonstrated that Fer-1 can also alleviate ferroptosis, which inhibits the release of HMGB1 and the subsequent inflammatory response, thus exerting a protective effect in PH. The ROS generated by ferroptosis can regulate the expression of endothelial to mesenchymal transition (EndMT)-related genes via the ROS-TGF-β axis, thereby endowing endothelial cells with the characteristics of mesenchymal cell migration and invasion capabilities, and thus promoting vascular remodeling (80).

**Figure 1:**
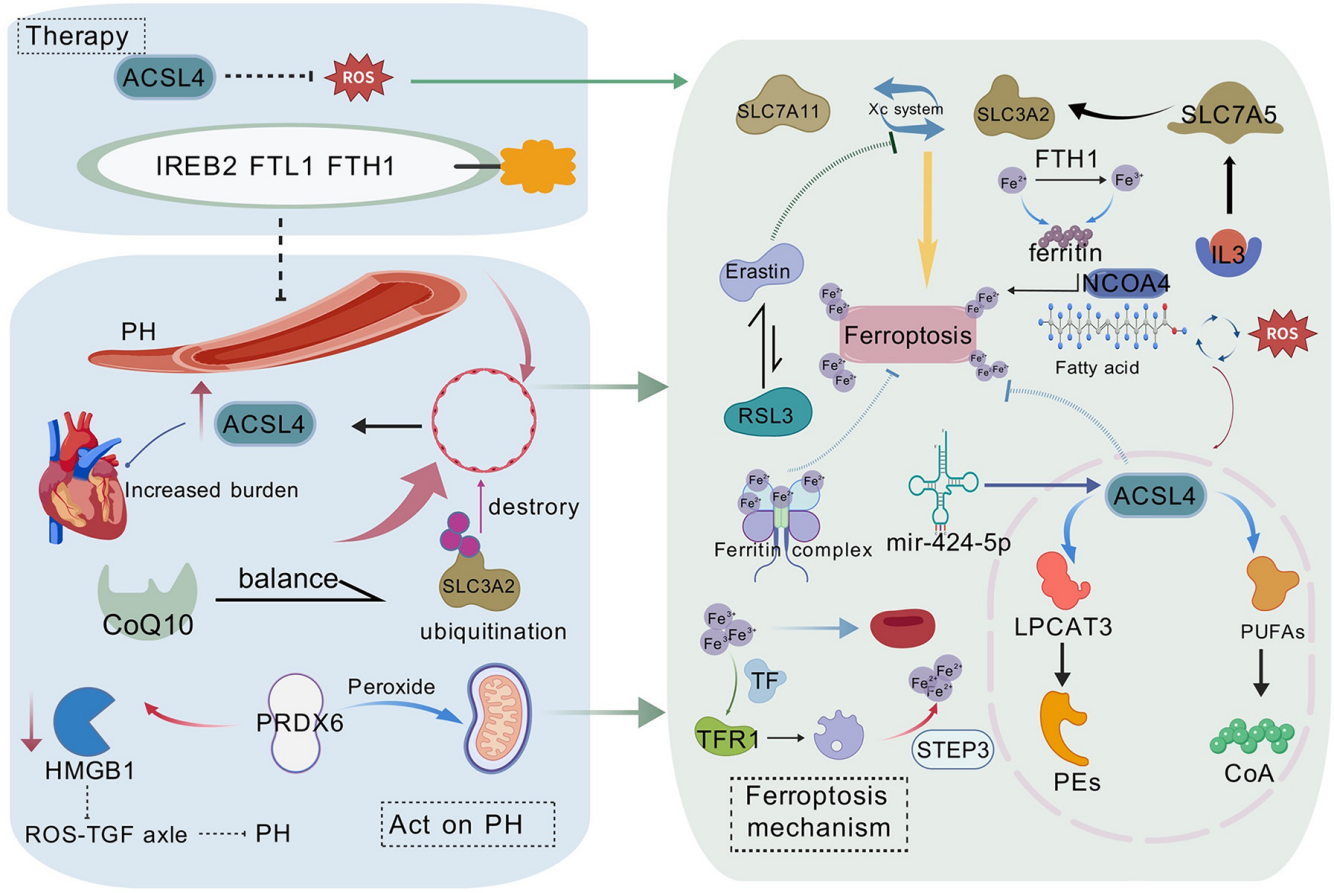
The molecular mechanisms of ferroptosis in pulmonary hypertension (PH) and potential therapeutic strategies are illustrated. PH affects endothelial cells, leading to lipid peroxidation and ferroptosis. ACSL4 plays a central role in lipid peroxidation, whereas iron metabolism disorders promote ferroptosis by affecting ferritin (FTL1/FTH1) and ferroportin (FPN1). The figure also presents potential therapeutic strategies targeting the Xc^−^ system (including SLC7A11 and SLC3A2), ACSL4, and ferroptosis suppressor protein 1 (FSP1), offering new perspectives and therapeutic avenues for PH treatment. This figure is based on Bio Gdp.

Moreover, maintaining an appropriate level of Coenzyme Q10 (CoQ10) is crucial for protecting pulmonary cells. FSP1 synergizes with GPX4 to inhibit ferroptosis (20). Chen et al. (81) showed in aortic vessels that blocking the FSP1-CoQ10 pathway reduces cell viability and increases lipid peroxidation levels associated with ferroptosis in smooth muscle cells, exacerbating inflammatory damage, suggesting that the FSP1-CoQ10 pathway's role in counteracting ferroptosis may be significant in pulmonary vascular pathology. Rutin, a naturally occurring flavonoid, protects PAH rats from ferroptosis damage by directly interacting with protein kinase C (PKC)α and altering its structure and activity (82), highlighting the therapeutic potential of small and large molecule interactions. These findings not only provide new insights into PH pathogenesis but also offer potential interventions and therapeutic targets (Figure 1).

### 4.2 Cuproptosis as a potential therapeutic target in PH

Cu, an essential trace element, may exacerbate PAH progression, with metabolic factors likely playing a dominant role. Cu affects vascular endothelium and smooth muscle, is associated with vascular aging, and participates in energy metabolism, signal transduction, and biomolecule synthesis (83, 84). Endothelial cell-derived nitric oxide (NO) diffuses to pulmonary smooth muscle cells (PSMCs) to induce vasodilation and oppose vascular remodeling. Cu contributes to NO synthesis and release, and restoring NO levels can prevent EndMT in PAECs, thereby counteracting the endothelial-to-mesenchymal transition that promotes a proliferative phenotype (85). Thus, inhaled nitric oxide (iNO) therapy effectively reduces PVR but is less effective in advanced stages, which are often accompanied by right heart failure (86). Moreover, a recent report showed that in children with PH who experienced in-hospital cardiac arrest, iNO treatment was associated with a lower uncorrected return of spontaneous circulation rate than that in controls (56), highlighting the need for further investigation of PH treatment.

**Figure 2:**
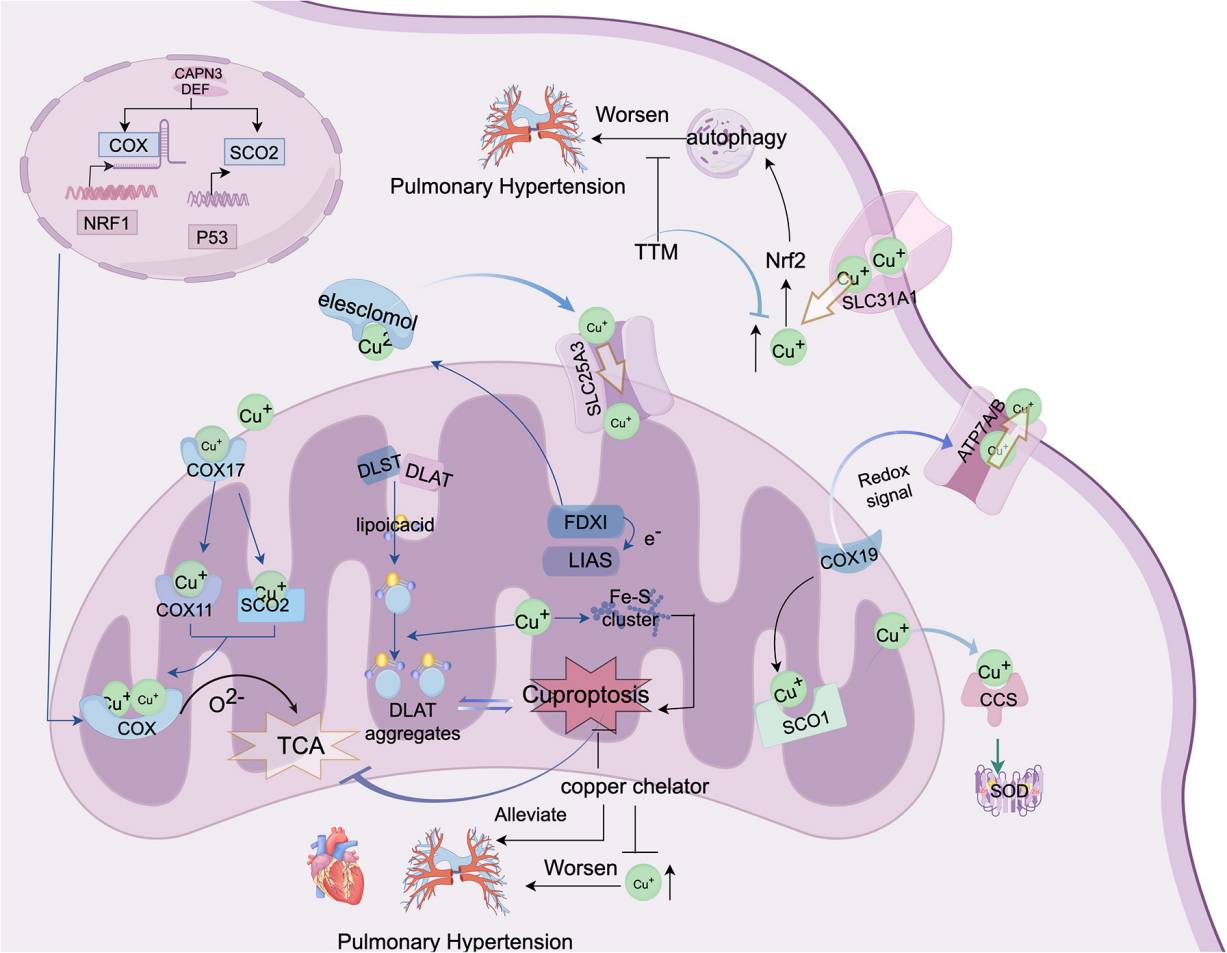
This figure depicts the molecular mechanisms of cuproptosis and potential therapeutic strategies for PH. This shows how abnormal copper metabolism and cuproptosis may contribute to PH progression. Cytochrome c oxidase (COX) and lipoic acid-containing proteins are the central components of copper metabolism, both localized in the mitochondria. The figure also demonstrates that copper can directly bind to lipoic acid components to induce cuproptosis. Additionally, elevated copper levels may exacerbate pulmonary vascular remodeling by activating autophagy/cuproptosis, thus offering new insights and potential therapeutic avenues for PH treatment. The figure was created using FigDraw. ID: AYOYOFCAAB.

Animal studies have shown that Cu overload induces mitochondrial abnormalities and inhibits angiogenesis, ultimately leading to Cuproptosis. Mo et al. (87) demonstrated that chelator-mediated reduction of Cu²^+^ in rat cerebral vessels inhibited cuproptosis, improved blood perfusion, and alleviated mitochondrial damage. Additionally, data from Phase II clinical trials indicated that the Cu ion chelator triethanolamine reduces Cu²^+^ toxicity on mitochondrial function and energy metabolism, significantly improving left ventricular systolic function (88). This also implies the potential involvement of cuproptosis in the pulmonary vasculature. However, direct studies linking cuproptosis to PH are lacking, although evidence suggests that PH severity correlates with elevated Cu ion levels in pulmonary vascular cells, possibly because of hypoxia or other factors upregulating Cu transporters (89). Excessive Cu accumulation may exacerbate PH progression by promoting cell death. Cu chelators have been shown to inhibit PAEC proliferation in patients with idiopathic pulmonary arterial hypertension (IPAH) by activating Apoptosis-Inducing Factor protein and triggering a non-caspase-dependent apoptotic pathway (90). Moreover, a diet deficient in Cu will not cause further damage to the right atrium (90, 91). These findings suggest that the role of Cuproptosis in PH and the activation or inhibition of associated signaling pathways in pulmonary vascular cells represent crucial directions for future research (Figure 2).

### 4.3 Therapeutic potential of synergistic cuproptosis and ferroptosis in clinical and animal studies

Aberrant Cu and Fe metabolism has been implicated in the pathogenesis of cardiovascular diseases (92). In the context of pulmonary vascular remodeling, exploring the interplay between these two forms of cell death may offer a more effective strategy for mitigating PH progression. However, current research on the synergistic effects of ferroptosis and cuproptosis remains largely limited to preclinical studies, with nanotechnology showing promising results. For instance, the MitCuOHA nanozyme has been shown to deplete cysteine (Cys), release Cu ions, and induce lipoylated protein aggregation, thereby simultaneously triggering cuproptosis and ferroptosis both *in vitro* and *in vivo*, resulting in enhanced tumor growth suppression (93). Similarly, Erastin and Cu—conjugated nanoparticles deplete glutathione (GSH), increase lipid peroxidation, inhibit the TCA cycle, and promote T-cell infiltration (16, 94). A nanocarrier system responsive to GSH via disulfide bonds (-S-S-) was also found to inhibit GPX4 and induce DLAT oligomerization, ultimately activating both cuproptosis and ferroptosis in tumors (95). Moreover, in treating myelodysplastic syndromes, combining the ferroptosis inducer imidazole ketone erastin (IKE) with Elesclomol-Cu (ES-Cu) induces severe mitochondrial damage, elevates ROS levels, and enhances lipoylation-dependent DLAT oligomerization, resulting in more pronounced cell death and greater inhibition of proliferation compared to monotherapy (96). Likewise, compared to the toxic effects of Cu chelation therapy, the traditional Chinese medicine curcumin has demonstrated marked efficacy in Wilson's disease by lowering intracellular Cu levels, inhibiting ferroptosis, and promoting the expression of oxidative stress-related markers such as GPX4, heme oxygenase 1 (HO-1), and Nrf2 (97). These findings underscore the therapeutic potential of co-targeting ferroptosis and cuproptosis in disease treatment. Nevertheless, clinical reports on the combined induction of these two forms of cell death remain scarce.

Although research on the interplay between cuproptosis and ferroptosis has primarily focused on cancer, a notable gap remains in cardiovascular research. Cancer cells often exhibit a Warburg (glycolytic) phenotype to meet excessive growth demands. In mouse models of early-stage PH, reduced activity of electron transport chain complexes in pulmonary endothelial cells elevates mitochondrial ROS, which upregulates hypoxia-inducible factor-1 alpha (HIF-1α) and enhances glycolysis. This results in a metabolic profile resembling that of cancer cells—albeit without invasion or metastasis—ultimately contributing to vascular remodeling (98, 99). These observations suggest that insights from cancer research on the interaction between cuproptosis and ferroptosis may also be relevant to pulmonary vascular remodeling.

## 5 Crosstalk between cuproptosis and ferroptosis in different types of PH

**Figure 3:**
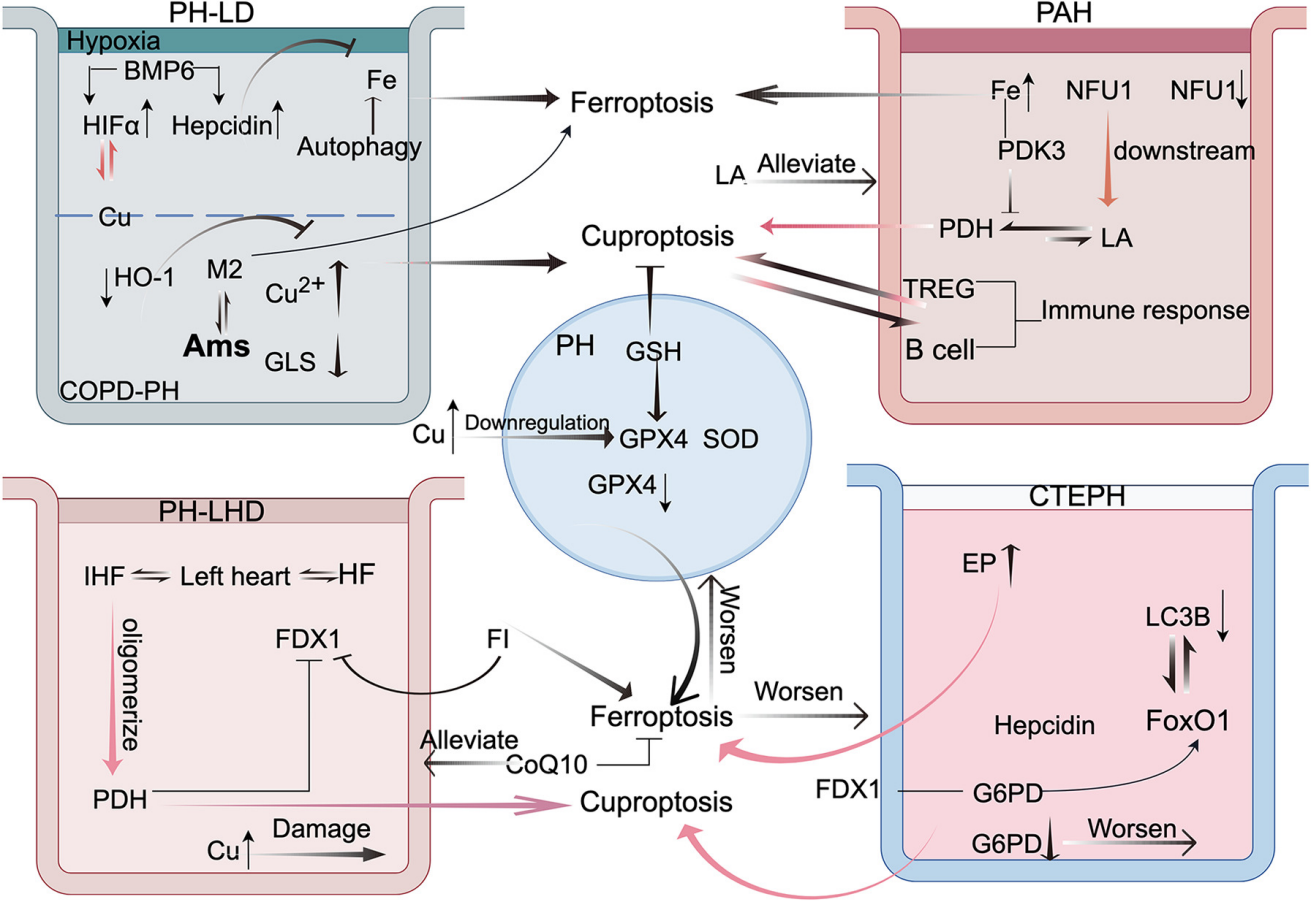
This figure illustrates the crosstalk between cuproptosis and ferroptosis in the four types of pulmonary hypertension (PH). This highlights the mechanisms of interaction between these two forms of cell death in pulmonary disease-associated PH, emphasizing the roles of hypoxia-inducible factor-1 alpha (HIF-1α) and alveolar macrophages (AMs). In arterial PH, immune responses and pyruvate dehydrogenase (PDH) are central to the crosstalk. In PH because of left heart disease, frataxin (FDX1) is the key mediator. In chronic thromboembolic pulmonary hypertension, glucose-6-phosphate dehydrogenase (G6PD) and forkhead box protein O1 (FoxO1) play pivotal roles. These findings offer new perspectives and potential therapeutic avenues for PH treatment. FI: ferroptosis inducer. This figure was created based on FigDraw. ID: OIPSOe5796.

As more forms of regulated cell death are being discovered, a strong correlation between ferroptosis and cuproptosis has become increasingly evident (100). Similarly, the dysregulation of Fe and Cu homeostasis plays a significant role in the pathogenesis of PH (27, 28, 70) (Figure 3). In PH, the diminished antioxidant capacity of pulmonary vascular cells exacerbates the insufficient GPX4 supply, which in turn promotes oxidative stress and elevated levels of lipid peroxidation (101). Recent studies have shown that Cu can directly bind to GPX4, inducing its oligomerization, which is subsequently degraded by the autophagy receptor Tax1 Binding Protein 1 during autophagy (102), thereby driving ferroptosis. 4-HNE, as a marker of ferroptosis, can also covalently bind to and inactivate the GPX4 protein (103) and inhibit antioxidant systems such as SOD and Glutathione Peroxidase(GPX), leading to cellular oxidative damage and activation of stress signaling pathways (104–106). GSH serves as a cofactor for GPX. It can not only inhibit lipid peroxidation to block ferroptosis but has also been found in pancreatic cancer tissues, where it can act as a ferroptosis inhibitor. This may involve the increased transport of GSH into the mitochondria to exert its effects via solute carrier family 25 member 39 (SLC25A39) (107), whereas SLC25A3 facilitates the transport of Cu into the mitochondrial matrix for enzymatic use (108). These findings highlight the pivotal role of GSH in the regulatory networks of cuproptosis and ferroptosis in PH.

### 5.1 PH-LD

PH associated with chronic pulmonary disease often exhibits unique pathological characteristics, primarily involving the loss of small pulmonary vessels, leading to vascular remodeling and increased mortality. However, the efficacy of conventional pharmacological treatments for PH remains limited (109), highlighting the urgent need for further research.

#### 5.1.1 High-altitude pulmonary hypertension (HPH)

In the development of HPH, it is proposed that under hypoxic conditions, the hypoxia-induced mitochondrial autophagy receptor FUN14 domain containing 1 (FUNDC1) modulates HIF1α activity, thereby stimulating pulmonary artery smooth muscle cells (PASMCs) proliferation and triggering PH (110). In hypoxic PH rats, both hepcidin and HIF1α levels were elevated via the mediation of bone morphogenetic protein 6 (BMP6). BMP6 regulates systemic Fe levels, whereas hepcidin reduces intracellular Fe release (111). Consistent with this, Hu et al. observed that during early hypoxia, ferroptosis and ferroptosis resistance coexist, with PASMCs proliferation outweighing cell death, This may result from low pulmonary artery Fe levels and ROS accumulation, enhancing cellular defenses (112). Similarly, clinical reports indicate that patients with Fe deficiency exhibit higher rates of cardiovascular mortality and heart failure (113). In this context, ferroptosis inhibition paradoxically exacerbates PASMCs proliferation. However, when ferroptosis is induced via autophagy, such as through the induction of the long non-coding RNA MIR210HG, it can aggravate HPH by promoting a synthetic phenotype in PASMCs (114). Therefore, maintaining a balance between ferroptosis and proliferation is crucial in hypoxic PH. Additionally, in tumor models, MURR1 domain upregulation in Cu metabolism reduces Cu content, promotes ubiquitin-mediated HIF1α degradation, and downregulates CP and SLC7A11 transcription, thereby enhancing ferroptosis (115). Moreover, during cuproptosis induction, SLC7A11 degradation also occurs, triggering severe oxidative stress and lipid peroxidation (116), thereby providing further evidence of a potential link between these two forms of cell death. Thus, the interaction between ferroptosis and cuproptosis can be mediated through HIF1α, although research on the role of Cu in hypoxic pulmonary vasculature remains limited.

#### 5.1.2 COPD-PH

COPD is one of the most common diseases worldwide and is characterized primarily by chronic inflammation and destruction of the lung tissue. Pulmonary vascular remodeling can be an early pathological change that is highly likely to induce irreversible emphysema. As the disease progresses, the accumulation of inflammation may trigger cardiovascular diseases, such as heart failure and PH (117). Smoking is the primary etiological factor. Recently, it was discovered that in patients with COPD, overexpression of fibroblast growth factor (FGF) 10 not only reversed cigarette smoke-induced emphysema but also exerted a protective effect on the pulmonary vasculature, preventing the development of PH (118). In pulmonary tissues, FGF 10 exerts its effects via the Nrf2-mediated antioxidant pathway, which activates the downstream SLC7A11/GPX4 axis. This process reduces free Fe and lipid peroxidation, thereby alleviating ferroptosis and ROS (119). These findings suggest that ferroptosis is involved in COPD-associated PH. However, excessive activation of Nrf2 or exposure to cigarette smoke can over-activate heme oxygenase 1 (HO-1), which catalyzes heme to release large amounts of Fe²^+^. This can increase Fe-dependent lipid peroxidation and potentially cause ferroptosis-related lung tissue damage (120). However, the biliverdin generated in this process exhibits antioxidant properties. Recent studies have suggested that HO-1 can exert antioxidant effects in alveolar cells, which are regulated by the glycoprotein CEACAM6, a member of the carcinoembryonic antigen family. When high levels of CEACAM6 inhibit HO-1, it fails to resist the oxidative damage caused by cigarette smoke, thereby potentially promoting COPD development (55). These findings highlighted the dual role of HO-1.

In COPD, alveolar macrophages (AMs) are the predominant immune cells and are classified into two phenotypes: M1 and M2. The proinflammatory M1 phenotype promotes lipid peroxidation and ferroptosis in alveolar cells via the paracrine secretion of LTB4, thereby facilitating COPD progression (121). The anti-inflammatory M2 phenotype is susceptible to ferroptosis and consequently loses its anti-inflammatory function, exacerbating the inflammatory response and lung injury. Thus, HO-1 inhibition can reverse the inflammatory damage caused by ferroptosis in AMs (122). Nevertheless, M2 macrophages, often implicated in abnormal tissue repair, not only secrete fibrotic factors that promote airway remodeling in patients (123), but also stimulate the proliferation of PASMCs via secretion of the chemokine CX3CL1 (124). However, recent findings indicate that, in the context of smoke exposure alone, the absence of inducible nitric oxide synthase—commonly expressed in macrophages—can prevent the pro-proliferative effects of M2 macrophages on PASMCs. This may involve the extracellular signal-regulated kinase (ERK) signaling pathway and contribute to pulmonary vascular remodeling in patients with COPD (125). Additionally, in a COPD rat model, AMs showed decreased cuproptosis-related gene glutaminase (GLS) and increased Cu²^+^. Because GLS converts glutamine to glutamate during the TCA cycle, its reduction may cause energy deficits in AMs, inducing the release of inflammatory factors and further lung damage (126). Notably, plastic particles entering AMs can also increase SLC31A1 expression, promoting cuproptosis and tumor necrosis factor-α secretion, which activates alveolar epithelial inflammation and destroys lung tissue (127). Moreover, exposure to cigarette smoke can activate a large number of macrophage infiltrations within blood vessels, triggering oxidative stress and endothelial dysfunction, which, in turn, exacerbates PH (128). Cigarette smoke extract can elevate the levels of superoxide anions (O₂^−^) in PASMCs (129). This leads to a paradoxical decrease in the levels of nitric oxide (NO) generated by the catalytic action of nitric oxide synthase, thereby impeding the vasodilatory effect of NO on PASMCs (130). However, in lung tissue, excessive NO can react with O₂^−^ to generate the potent oxidant peroxynitrite (ONOO^−^), further exacerbating COPD-associated damage. In conclusion, in the context of AMs, the concurrent occurrence of cuproptosis and ferroptosis has been implicated in the worsening of COPD progression. Nevertheless, the role of cuproptosis/ferroptosis in macrophages, particularly M2 macrophages, in pulmonary vascular remodeling requires further investigation.

Furthermore, experimental validation has shown that circSAV1 promotes the translation of IREB2 mRNA through m6A modification, leading to Fe overload and ferroptosis, thereby inducing COPD. IREB2 binds to Fe-responsive elements (IREs) to maintain Fe homeostasis (131). When Fe levels are low, Fe regulatory protein 1 loses its Fe-S cluster and binds to IREs in target mRNAs to promote Fe uptake and reduce Fe storage and utilization (132). Upregulation of the Fe-S cluster assembly protein IscU2 can inhibit this process because IscU2 is a scaffold protein that stabilizes Fe-S cluster-containing proteins. In pancreatic cancer tissues, IscU2 enhances the expression of the cuproptosis-related factor DLST during the TCA cycle (133). Additionally, researchers have discovered that the human Fe-S cluster assembly enzyme (ISCU) protein, owing to its strong Cu-binding activity, can hinder the assembly of Fe-S clusters. In Menkes disease, as Cu accumulates, the activities of alpha-ketoglutarate dehydrogenase (KGDH) and PDH decrease, both of which require Fe-S clusters (134). Collectively, these findings suggest that Fe-S clusters may serve as regulatory switches in the interactions between Cu and Fe metabolism.

Moreover, clinical investigations have revealed that the degree of pulmonary fibrosis is associated with the risk of mortality in PH (135). Furthermore, fibroblasts and PASMCs exhibit excessive resistance to cell death and hyperproliferation (136). Both Cu and Fe overload result in the accumulation of reduced ferrous and cuprous ions, initiating the Fenton reaction and generating large amounts of ROS (137). ROS induce TGF-β1, promoting fibroblast activation and extracellular matrix deposition, thereby exacerbating pulmonary fibrosis. Simultaneously, TGF-β1 increases Fe accumulation and induces ferroptosis (138). In summary, in pulmonary diseases, HIFα, AMs, and Fe-S clusters serve as hubs linking cuproptosis and ferroptosis, potentially emerging as new research hotspots in the future.

### 5.2 PAH

PAH is a vascular dysfunction associated with inflammatory infiltration and is closely related to mitochondrial dysfunction (139). For instance, studies have shown that serum ceruloplasmin (CP) levels are significantly elevated in patients with systemic sclerosis-associated PAH and PAH mouse models (140). CP is a vital protein responsible for binding Cu and transporting it to target organs. It relies on ferroxidase (FOX) activity to oxidize Fe2 + to Fe3+, thereby facilitating Fe transport. Excess Cu in the liver promotes FOX synthesis, potentially enhancing Fe absorption (141). Elevated Cu levels in the body can also induce oxidative stress and vascular dysfunction, both of which are detrimental to cardiovascular health (142).

When superoxide exacerbates endothelial injury, it contributes to the pathogenesis of PAH, as evidenced by the decreased levels of the antioxidant enzyme superoxide dismutase (SOD) in idiopathic PAH (143). Additionally, cohort studies have suggested that this disease is closely linked to autoimmunity, characterized by abnormal immune cell infiltration (144), including increased regulatory T-cell (Treg) concentrations and B cell frequencies. In hereditary PAH (HPAH) patients, the severity of pulmonary vascular lesions is associated with TIM-3-positive T cells (92). Although immune inflammation is a common feature of this disease, the precise relationship between immunity and IPAH remains unclear. Cuproptosis induces immunogenic cell death, thereby activating numerous immune cells (145). Similarly, Fe accumulation activates immune cells (146), both of which contribute to inflammatory responses. Cheng et al. (57) reported that lung cancer exhibits pulmonary vascular remodeling features of PH, possibly because of tumor cell-induced inflammation affecting the pulmonary vessels. Therefore, the immune effects of cuproptosis and ferroptosis may provide new perspectives for treating PH.

Cuproptosis, ferroptosis, and metabolism are closely linked to mitochondrial damage and cellular respiration (147). NFU1, a mitochondrial Fe-S scaffold protein, assembles and transfers Fe-S clusters to target proteins such as complex II and LIAS. Mutations in NFU1 impair these electron transport proteins, weaken mitochondrial respiration, and promote pulmonary artery smooth muscle cell proliferation (148). Accordingly, humanized NFU1 mutations leading to mitochondrial dysfunction are closely associated with pulmonary vascular remodeling, with a marked NFU1 deficiency observed in patients with IPAH. Supplementation of the downstream target LA in PAECs from patients with PAH has been shown to improve mitochondrial function (149). High Cu concentrations can also compromise the mitochondrial membrane (150). LA is a component of the PDH complex, and lipoyltransferase 1 (LIPT1), a recently identified cuproptosis-related gene, transfers LA from glycine cleavage system protein H to the E2 subunit of lipoylated substrates, thereby influencing PDH activity, as observed in cancer and metabolic studies (151–153). Further investigation revealed that elevated Fe levels may bind to pyruvate dehydrogenase kinase (PDK) 3, inhibiting PDH complex activity (154). Clinically, a 4-month trial demonstrated that administration of the PDK inhibitor dichloroacetate to patients with IPAH activated PDH, reduced PVR, and improved lung function (155). Conversely, another study found that PDK4-mediated inhibition of PDH could reduce pyruvate oxidation, thereby decreasing ROS generation in the TCA cycle, limiting lipid peroxidation, and mitigating ferroptosis (29). Thus, targeting mitochondrial ROS reduction may alleviate PDH-mediated ferroptotic damage and curb abnormal PASMC proliferation. Additionally, LIPT1 participates in the lipoylation of the mitochondrial enzymes PDH and KGDH. Studies on fibroblasts from patients with LIPT1 mutations have shown markedly reduced activity of these enzymes and intracellular Fe accumulation. In this setting, α-LA, an Fe chelator, inhibits ferroptosis and modulates PDH activity (85, 156). Consequently, suppressed PDH by iron metabolism may contribute to both cuproptosis and the inflammatory response, leading to adverse pathological outcomes.

### 5.3 PH-LHD

In left heart disease, the loss of cardiac function leads to reduced myocardial contractility, ultimately resulting in heart failure (HF). This condition increases PVR and decreases pulmonary arterial compliance, thereby inducing postcapillary pulmonary hypertension (pcPH). HF is the primary cause of pcPH, with ischemic heart disease (IHF) being a major contributor to HF. IHF results from insufficient coronary blood supply, which initially damages the left ventricular cells (157, 158). Excessive Cu ion levels in patients with HF induce oxidative stress and severe cardiac injury (159). Furthermore, cuproptosis has been implicated in IHF and is characterized by the oligomerization of PDH complex proteins. In response, the body initiates a self-defense mechanism by downregulating FDX1 expression. Moreover, a strong positive correlation has been observed between cuproptosis-related genes and Treg cells, which can promote cardiac remodeling in IHF through inflammatory pathways (160). In hepatocellular carcinoma studies, ferroptosis inducers have been shown to inhibit FDX1 degradation while reducing the Cu chelator GSH, leading to the accumulation of lipoylated proteins (161) and, ultimately, cuproptosis. Ferroptosis is widely associated with various cardiac diseases (162). CoQ10, a core electron carrier in the mitochondrial respiratory chain, exerts its antioxidant effects by inhibiting lipid/DNA oxidation, thereby suppressing ferroptosis. Clinical trials have demonstrated that high-dose CoQ10 improves hepatic steatosis and enhances cardiovascular function (163). In a study by Sharp et al., patients with PAH treated with CoQ10 exhibited increased hemoglobin and mean corpuscular hemoglobin levels, along with improved right and left ventricular function (164). Therefore, ferroptosis regulated by CoQ10 may play a significant role in left-sided heart disease. Thus, FDX1 may serve as a hub for these two types of cell death. However, this requires further investigation.

### 5.4 CTEPH

CTEPH often develops in patients following pulmonary embolism. Despite at least 3 months of anticoagulation therapy, thrombi in the pulmonary artery become organized and resistant to conventional anticoagulation, resulting in vascular obstruction, impaired blood flow, and elevated pulmonary arterial pressure, typically presenting as dyspnea (165). Increased erythrophagocytosis in pulmonary microvascular endothelial cells enhances procoagulant activity by inducing ferroptosis, thereby contributing to thrombotic vascular remodeling (166). PAEC dysfunction caused by thrombi represents a key pathological feature of CTEPH. Experimental studies have demonstrated impaired autophagic activity in PAECs of CTEPH rats, as indicated by the reduced expression of microtubule-associated protein 1 light chain 3 (LC3B), a protein involved in autophagosome formation (167). Forkhead box O1 (FOXO1), a member of the FOXO family, interacts with LC3 to regulate autophagy. FOXO1, a glucose regulator, has been shown to activate hepatic hepcidin transcription (168), thereby reducing Fe release. Bioinformatic data from studies on FOXO signaling indicate that glucose-6-phosphate dehydrogenase (G6PD), a key enzyme in the pentose phosphate pathway, is central to cellular processes in lung cancer tissues (169). During PH progression, G6PD mutations drive metabolic reprogramming and inflammation, thereby promoting pulmonary arterial remodeling (170). Animal studies have revealed that under conditions of Cu overload, G6PD binds to FDX1, reducing its stability and downregulating NADPH and GSH levels, thereby enhancing cuproptosis (171). Cu chelators also reduce lipid peroxidation levels; therefore, FOXO1 and G6PD may mediate the interaction between Cu and ferroptosis in CTEPH, which requires further study using CTEPH models. In summary, future research on cuproptosis and ferroptosis may offer valuable insights into improving patient survival.

## 6 Future perspectives

With a deeper understanding of ferroptosis and its mechanisms, the targeted regulation of cell death offers a novel direction for the treatment of PH. Although significant progress has been made in PH classification and therapy, researchers continue to identify new biomarkers and targeted drugs to improve diagnostic precision and slow disease progression. For instance, sotatercept, a BMP/TGF-β inhibitor, improves patient outcomes but may elevate heme oxygenase-1 levels, increase free Fe, and raise inflammatory mediator interleukin levels (172, 173). Given the complexity of the disease, the chronic nature of treatment, and the substantial economic burden, PH remains a highly challenging condition. Therefore, the distinct mechanisms of ferroptosis and cuproptosis may provide promising therapeutic avenues for PH and other diseases, thereby offering a theoretical basis for the development of new drugs and treatment strategies. Future research should focus on (1) further elucidating the mechanisms and pathophysiological roles of ferroptosis and cuproptosis in PH, particularly regarding mitochondrial respiration and oxidative stress, to address the current gaps in clinical and experimental evidence; and (2) examining the interplay between ferroptosis and cuproptosis, as well as the associations among different forms of cell death in various PH subtypes. These investigations will not only enhance our understanding of PH pathophysiology but also yield new perspectives for its treatment.
